# New Aspects on the Direct Solid State Polycondensation (DSSP) of Aliphatic Nylon Salts: The Case of Hexamethylene Diammonium Dodecanoate

**DOI:** 10.3390/polym13162625

**Published:** 2021-08-06

**Authors:** Angeliki D. Mytara, Athanasios D. Porfyris, Stamatina N. Vouyiouka, Constantine D. Papaspyrides

**Affiliations:** Laboratory of Polymer Technology, School of Chemical Engineering, National Technical University of Athens, Zographou Campus, 15780 Athens, Greece; amytara@mail.ntua.gr (A.D.M.); adporfyris@mail.ntua.gr (A.D.P.); mvuyiuka@central.ntua.gr (S.N.V.)

**Keywords:** aliphatic polyamides, direct solid state polymerization, polycondensation water, polymerization at the microscale

## Abstract

The direct solid state polymerization (DSSP) of hexamethylene diammonium dodecanoate (PA 612 salt) was investigated for two different salt grades, fossil-based and bio-based. Aliphatic polyamide salts (such as PA 612 salt) are highly susceptible to solid melt transition (SMT) phenomena, which restrain the industrial application of DSSP. To that end, emphasis was given on reactor design, being the critical parameter influencing byproduct diffusion, amine loss and inherent DSSP kinetics. Experiments took place both at the microscale and the laboratory scale, in which two different reactors were tested in terms of bypassing SMT phenomena. The new reactor designed here proved quite successful in maintaining the solid state during the reaction. Scouting experiments were conducted in order to assess the effect of critical parameters and determine appropriate reaction conditions. Fossil-based PA 612 products proved to have a better end-group imbalance in comparison to bio-based ones, which is critical in terms of achieving high molecular weight. Finally, a real DSSP process was demonstrated, starting from PA 612 salt crystals and ending with PA 612 particles.

## 1. Introduction

The direct solid state polymerization (DSSP) of polyamide salts has been systematically investigated in our laboratory since the late 1970s [1,2,3,4,5,6,7,8,9,10,11,12,13,14,15,16,17,18,19,20,21,22,23,24,25,26,27,28,29,30]. DSSP involves heating the solid reacting mass at a temperature below its melting point and the melting point of the final polymer product under an inert atmosphere as to allow the progression of the polycondensation, while maintaining the solid phase in the reacting mass. DSSP has been found to yield high quality products as side reactions and thermal degradation are avoided. The reason for this lies mainly in the low reaction temperatures applied. Thus, DSSP is the most appealing candidate for industrial application due to the mild reaction conditions (in terms of pressure and temperature), the absence of solvents and the simple equipment used. This renders the process both cost and environmentally friendly, especially since the products obtained present high conversion and excellent properties for relatively short reaction times [12,17,24,26,28,29,30].

Numerous studies have appeared in the open and patent literature on the DSSP of polyamide salts. Polyamide salts combine both acid and amine groups and usually can crystallize from a solution [7,10,11,25,29,31]. Papaspyrides et al. have studied the DSSP of PA 126 salt (nylon^®^ 126 salt) at a temperature range up to 25 °C below its melting point. The salt was suspended in an inert non-solvent and a distinct transition to the melt state and agglomeration of the solid crystals was clearly observed thanks to the glassware assembly employed [2,3]. The same behavior was noticed later in the same laboratory for a series of aliphatic nylon^®^ salts including PA 26, PA 210, PA 46, PA 66, PA 410, PA 610 and PA 1210 [7,8]. Conversion before melt occurrence remained particularly low (approximately at 0.05–0.1), clearly indicating that it is impossible to achieve polymer formation, while maintaining the solid state [7].

DSSP kinetics (in the stage prior to transition to the melt) fit well typical nucleation and growth data. The nucleation stage occurs first on the surface of the crystallites and then in the defective sites. In parallel it has been confirmed that during the initial stages of the DSSP of polyamide salts, the volatile diamine component escapes in both aliphatic (nylon^®^) salts and semi-aromatic polyamide salts [20,28,29,32,33]. In addition, in a DSSP study on PA 66, it was discovered that diamine loss preceded water formation, thus resulting in even more defects in the salt crystal lattice, which in turn are active centers for initiation and propagation [20].

Following nucleation, the growth stage often proceeds unexpectedly. The transition to the melt state or the so-called solid melt transition (SMT) has been linked with the formation of the polycondensation water in the reacting mass. Papaspyrides et al. in a series of studies proposed a mechanism for SMT as follows: water formed during the solid state polycondensation reaction hydrates the polar groups of the hydrophilic polyamide salt. As the reaction proceeds and the amount of polycondensation water increases, the crystal structure of the salt is destroyed by the formation of highly hydrated regions. These areas have a much lower melting point and soon fall in the melt state, resulting in the experimentally observed transition of the reaction from the solid to the melt state. As the reaction proceeds further, the molecular weight increases, the hygroscopicity of the reacting system decreases and finally the solid character of the system is restored [3,4,5,6,7,8,9,10,17,24].

Further studies revealed that the occurrence of SMT is highly dependent on the inherent chemical structure of the polyamide salt. The DSSP of ethylene diammonium fumarate, ethylene diammonium maleate and hexamethylene diammonium fumarate, which have a more organized and rigid network of polar coordinating groups than the aliphatic polyamide salts, presented severe deviations from the SMT model [7,9]. In these cases, water accumulation to the solid mass was noted along with a phase separation, thus indicating a link between the occurrence of SMT and the nature of the polyamide salt and its structural organization [9]. The latter point was highlighted also in recent studies on the DSSP of semi-aromatic PA 4T and PA 6T polyamides [28,30]. Within the experimental conditions applied, the reacting PA 4T and PA 6T salt crystals, maintained their morphology as a whole, and no agglomeration or sticking was evident, suggesting that the reaction could proceed in true solid state.

As aliphatic polyamides are of significant commercial and industrial interest, accounting for more than 40% of total polyamide demand, many attempts have been made to overcome, manage or even exploit the transition to the melt, aiming to open the route for the industrial application of DSSP. A series of organic and metal catalysts have been tested, both as to their influence on the reaction rate and on the occurrence of SMT [4,5,6,11,17,19,23,24,34]. The presence of an effective catalyst in the DSSP of both PA 126 salt and PA 66 salt, was found to favor the diffusion of polycondensation water and the reaction is shifted to the right, while bypassing SMT phenomena [4,5,11]. In particular for the DSSP of PA 66 salt effective catalysts include H_3_BO_3_, (COOH)_2_, H_3_PO_4_, and MgO. It was also found that the method applied for the incorporation of the catalyst in the reacting mass influenced the effectiveness of the catalytic action, as the coprecipitation of the catalyst along with the polyamide salt proved more effective than dry mixing, probably due to the ionic bonds of the catalyst with the polyamide monomer.

Turning to reactor aspects, it has been proposed the removal of the polycondensation water at atmospheric pressure, mentioning however that “the solid mass never completely liquifies”, suggesting that some degree of agglomeration and/or partial melting is involved [35]. Papaspyrides et al. proposed initially a pre-polymerization process in a typical high pressure batch autoclave. Dry PA 66 salt was heated under autogenous pressure in the proximity of its melting point to minimize the loss of hexamethylene diamine. The process was followed by a venting stage, in which pressure release allows water removal, shifting the reaction to the right [15,16]. Later an extrusion type assembly was employed for scaling up [14]. PA 66 salt was reacted slightly above its melting point, ca. 200 °C. The machinery used was capable of handling all process stages encountered, namely melting the dry salt fed, then reacting at the melt state and finally, while the mass was solidified, completion of the reaction in the solid state. In fact, as has been mentioned already when molecular weight increases the reacting mass starts solidifying again and this would inevitably result in severe damages on any conventional equipment employed [14].

On the other hand, SSP runs have been also carried out at the micro scale and in a TGA chamber. This route ensures runs are easy to make, and thus a wide variety of valuable kinetic data at different critical parameters, such as time, temperature and gas flow rates. Historically this effort has been originating in the sixties when Volokhina et al. polymerized nylon salts, such as PA 66 and PA 6T salt in a TGA “micro” reactor. These experiments were continued in 1990s, polymerizing PA 66 in the presence of boric acid as a catalyst. It was discovered that the presence of the catalyst changes dramatically the reaction mechanism so that in this case the reaction is not any more controlled by diffusion but by intrinsic kinetics [32,33]. The same conclusion was met later, studying the DSSP reaction of semi-aromatic polyamide salts but in this case even in the absence of catalysts [28,29]. Finally, it was also proved that when scaling up from the TGA micro reactor to a laboratory scale reactor, the same picture was encountered but when operated at the same critical reaction parameters [30].

In conclusion, the occurrence of SMT presents a major obstacle when considering the application of DSSP in an industrial scale at least for the production of aliphatic nylon^®^ salts. Moreover, catalysts negatively influence the purity of polyamides restricting their spectrum of applications. In fact, and to the best of our knowledge, there is no such an industrial application of a DSSP route on aliphatic polyamide salts. In our laboratory, long time effort has been spent on developing technology via which the DSSP of aliphatic nylon^®^ salts would proceed at an adequate rate and reach an acceptable conversion, while maintaining the solid state fully during the course of the reaction. As the diffusion of the polycondensation water has be proven to be very critical to the maintenance of the solid state, our interest has been focused also on reactor design to father facilitate byproduct removal.

In this paper hexamethylene diammonium dodecanoate (PA 612) was selected towards testing such a system for the following reasons: PA 612 belongs in a category of long aliphatic chain polyamides with increasingly extensive applications and commercial interest. The lower concentration of amide bonds in PA 612 compared with PA 6 and PA 66 is accounted for a number of properties desired in industry, such as low moisture sensitivity, combined with good dimensional stability, toughness and abrasion resistance, as well as relatively high melting point and good thermal stability [36,37,38,39,40,41]. On the other hand, the low hygroscopicity of this salt renders it a good candidate for DSSP. Beyond that, considering increasing academic interest towards bio-based polyamides from renewable sources, two grades of PA 612 salts were examined in this research, one fossil-based and one partly bio-based.

## 2. Materials and Methods

### 2.1. Starting Materials

The reactants used for the preparation of polyamide 612 (PA 612) were hexamethylenediamine (HMD, Alfa Aesar, Kandel, Germany) and dodecanedioic acid (DDDA). Two different grades of DDDA were used, one fossil-based grade purchased from Alfa Aesar (purity 99%) and a bio-based one provided by Cathay Biotechnology (Cathay, Biotechnology, Shandong, China) (purity ≥ 98.5%). Commercial polyamide 66 (PA 66) salt from BASF (BASF, Ludwigshafen, Germany) was used as reference material for characterization purposes. Ethanol (EtOH) by Acros Organics (Schwerte, Germany) was used as a solvent for the potentiometric titration of polyamide salts. O-cresol (Merck, Hohenbrunn, Germany), phenol (Chemlab, Zedelgem, Belgium), methanol (MeOH, Fischer, Kandel, Germany), 1,2 dichlorobenzene (ODCB) and lithium chloride (LiCl, Alfa Aesar) were used as solvent systems for potentiometric titration of polyamide samples. Sulfuric acid 99% (Fluka, Seelze, Germany) was used as a solvent for viscosity measurements.

### 2.2. Polyamide Salt Preparation

PA 612 salt was prepared as described in previous research of ours [29,31]. Specifically, 66.5 g (0.288 mol) solid DDDA were added to an aqueous solution of HMD (33.5 g, 0.289 mol) of a concentration of 10% *w*/*v*, and left to react at 50 °C under stirring for 1 h under reflux. The salt solution was then left to evaporate in a fume hood, washed with ethanol, dried in vacuo for 24 h (50 °C) and the final product was collected in the form of colorless crystals.

### 2.3. Direct Solid-State Polymerization (DSSP)

#### 2.3.1. Micro Scale (TGA) Runs

DSSP of PA 612 salt was first investigated at the micro scale, in a Mettler Toledo TGA/DSC 1 HT (Mettler Toledo, Columbus, OH, USA) instrument, using 100 μL aluminum crucibles to simulate SSP laboratory reactor conditions. Approximately 20 to 30 mg of dried PA 612 salt were placed in the crucible which was then sealed with a lid having a hole on the top, of 2 mm diameter. The samples were inserted into the TGA chamber at 30 °C (*T*_0_), preheated to 160 °C (*T*_1_) at a rate of 20 °C min^−1^, left at *T*_1_ for 15 min and further heated up to the nominal reaction temperature (*T*_DSSP_) at a rate of 1 °C min^−1^, followed by the final isothermal step at *T*_DSSP_ for periods ranging from 5 to 24 h (*t*_DSSP_). *T*_DSSP_ was chosen to be 20–30 °C below the melting point of the salt. Nitrogen was used as the inert gas at a constant flow rate of 25 mL min^−1^. After the isothermal step the material was cooled to room temperature. 

From the data obtained it is possible to calculate *t*_1/2_, which is defined as the time at which 50% of conversion is reached [28]. The total mass loss recorded from TGA experiments is compared to the theoretical loss of water of PA 612 salt polycondensation reaction, which can be calculated stoichiometrically (10.4% *w*/*w*), at conversion equal to 1. Amine loss is calculated as the deviation of the total mass loss from the theoretical one [28,29].

#### 2.3.2. Laboratory Scale Runs

Scaling up from micro scale DSSP experiments were carried out in two different laboratory scale reactors, namely Reactor 1 and 2. Reactor 1 is a commercial cylindrical autoclave by Parr Instrument Company (Parr Instrument Company, Frankfurt, Germany) (*d* = 3 cm, *h* = 17.5 cm), equipped with a gas inlet to permit preheated purge gas passing through the polymer mass during reaction. Reactor 2 is a fixed bed reactor more capable of removing the water formed. For each experiment, the reactor was filled with 10 g of dried PA 612 salt and purged several times with nitrogen as an inert gas at room temperature. Then nitrogen at 20 mL min^−1^ was set flowing through the salt and heating was started to the desired *T_DSS_*_P_. However, runs even at zero flow rates were carried out. After the end of the reaction (*t*_DSSP_ = 5 to 24 h), the reactor was cooled in an ice bath to *T*_cooling_ = 40 °C.

Finally, to demonstrate the feasibility of the DSSP process, the most promising product in terms of molecular weight and end group balance was selected and subjected to post-SSP. The reaction took place in a typical fixed bed reactor at 170 °C for 4 h. In this case, nitrogen was used only for purging and then flow rate was set at 0 mL min^−1^.

The nomenclature and DSSP reaction conditions all runs are presented in Table 1.

### 2.4. Characterization Techniques 

#### 2.4.1. Fourier Transform Infrared Spectroscopy (FT-IR)

Samples of both PA 612 salt and polymer were mixed with KBr crystals and then formed into pastilles. Spectra were recorded using a JASCO 4200 (JASCO, Gross—Umstadt, Germany) infractometer at frequencies from 4000 to 400 cm^−1^ with a resolution of 4 cm^−1^.

#### 2.4.2. Concentration of End Groups

End-group determination was achieved through potentiometric titration. The deviation of the mean values was derived through duplicate measurements. The titrant used for the determination of amine end groups was HClO_4_/MeOH (N = 0.1 meq mL^−1^) and for the carboxyl groups TBAH/BeOH (N = 0.05 meq mL^−1^). For the case of PA 612 salt 0.1% *w*/*v* solutions of salt in 70/30 EtOH/water were titrated. For the case of PA 612 oligomers/polymers, solutions of 0.5% *w*/*v* in Phenol/MeOH (90/10) for the determination of amine end groups and in o-cresol/ODCB (5%)//LiCl (20% in MeOH) (70/30) were used [21,22,25].

The experimental values for the polyamide salts were compared to the theoretical ones as calculated stoichiometrically.

#### 2.4.3. Solution Viscometry

The intrinsic viscosity [*η*] of synthesized polyamides was measured in sulfuric acid at a concentration of 0.5% *w*/*v* in a Cannon-Fenske viscometer (K= 0.2308 mm^2^ s^−1^) at 25 °C. The [*η*] value was obtained by the single point measurement applying Equation (1):

(1) $$\left[\eta \right]=\frac{\sqrt{1+1.5\;\eta_{sp}}-1}{0.75\;C}$$

where *C* (g mL^−1)^ is the solution concentration, and *η*_s*p*_ the solution specific viscosity [30,42]. The deviation of the mean values was derived through duplicate measurements.

#### 2.4.4. Thermal Properties 

DSC measurements were performed on a Mettler DSC 1 STAR System. Samples of 8–10 mg were placed in a 40 μL aluminum pan. PA 612 salt samples followed a single heating cycle from 30 °C to 280 °C with a heating rate of 10 °C min^−1^, while PA 612 products followed a heating–cooling–heating cycle from 30 °C to 280 °C with a heating and cooling rate of 20 °C min^−1^, under nitrogen flow (25 mL min^−1^). The melting point and specific heat of fusion of polyamides were obtained by data from the 2nd heating and the degree of crystallinity was calculated by Equation (2):

(2) $$X_{c}=\frac{\Delta H_{f}}{\Delta H_{o}}\%\;$$

where Δ*H_f_* (J g^−1^) is the experimental specific enthalpy of fusion and Δ*H_o_* (J g^−1^) is the specific enthalpy of fusion of the 100% crystalline polymer. For PA 612 this value is equal to 258 J g^−1^ [40,41,43]. 

TGA analysis was conducted in a Mettler Toledo TGA/DSC 1 HT thermobalance between 30–550 °C with a heating rate at 10 °C min^−1^, under nitrogen flow (25 mL min^−1^). Approximately 8–10 mg of each sample was placed in an alumina pan and then in the TGA chamber. Degradation temperature *T_d_* was obtained for the maximum mass loss rate. For PA 612 salt samples, the degradation temperature and residue at ca. 200 °C were obtained by data for the first mass loss “step” (step_1) [25]. The degradation at 5%, (*T_d,5%_*) was obtained for all PA 612 samples as the temperature where 5% of the initial mass is lost [29].

#### 2.4.5. Scanning Electron Microscopy

Scanning Electron Microscopy (SEM) was used to evaluate the morphology of the crystals. A Jeol 6300 JSM instrument was employed, with a high sensitivity secondary electron detector at 25 kV. The polyamide was coated with Au coating using a Quorum Technologies SC7620 sputter coater (Quorum Technologies, East Sussex, UK) with a sputtering time of 150 s at a current of 10 mA.

## 3. Results

### 3.1. Polyamide Salts Preparation

Two different grades of PA 612 salt derived from fossil- and bio-based DDDA were prepared, reaching a high mass yield (ca. 97%). The purity of the obtained salts and their end-group balance (*D* = [COO^−^] − [NH_3_^+^]) influence significantly the SSP polymerization degree that could be attained. Salts’ characterization data are presented in Table 2, and the measured amine and carboxyl group concentrations deviated slightly from the theoretical ones (5772 meq kg^−1^), with the highest deviation noticed for the case of the bio-based salt, probably due to impurities of the bio-based monomer. Both PA 612 salts are well balanced in terms of end groups though. Similar data were derived from the pertinent work of Boussia et al., in which, however, the PA 612 salt was formed via a different technology route, namely by mixing ethanolic solutions of the reactants at 0 °C [25]. It is also worth noting that the *D* values of all salts prepared in our laboratory were found much lower compared to a commercial PA 66 salt provided by BASF and used as a guiding reference.

**Figure 1:**
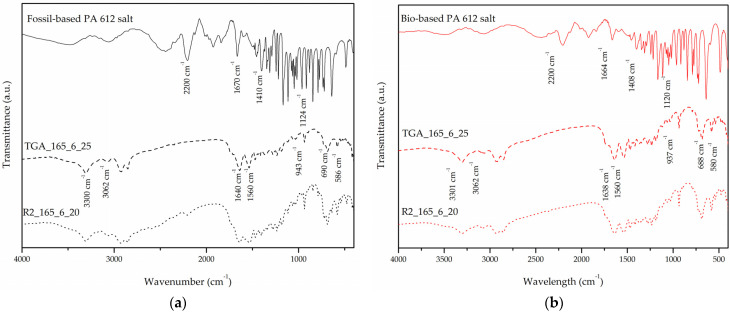
FT-IR spectra of the PA 612 salts and some characteristic DSSP products from both micro scale and laboratory scale runs. (**a**) Fossil- based grade and (**b**) bio-based grade.

The formation of a polyamide salt structure was confirmed through FT-IR analysis (Figure 1), as it was possible to identify peaks corresponding to ionized amine and carboxyl end groups. Specifically, the absorption at 2200 cm^−1^ is assigned to the stretching vibration of the ionized amine groups, while the absorption peak at 1410 cm^−1^ is assigned to symmetric stretching vibration of ionized carboxyl end groups. Furthermore, the weak absorption at 1670 cm^−1^ is attributed to NH_3_^+^ antisymmetric stretching vibration, whereas the weak absorption at 1124 cm^−1^ corresponds to NH_3_^+^ transverse rolling vibration [15,25].

**Figure 2:**
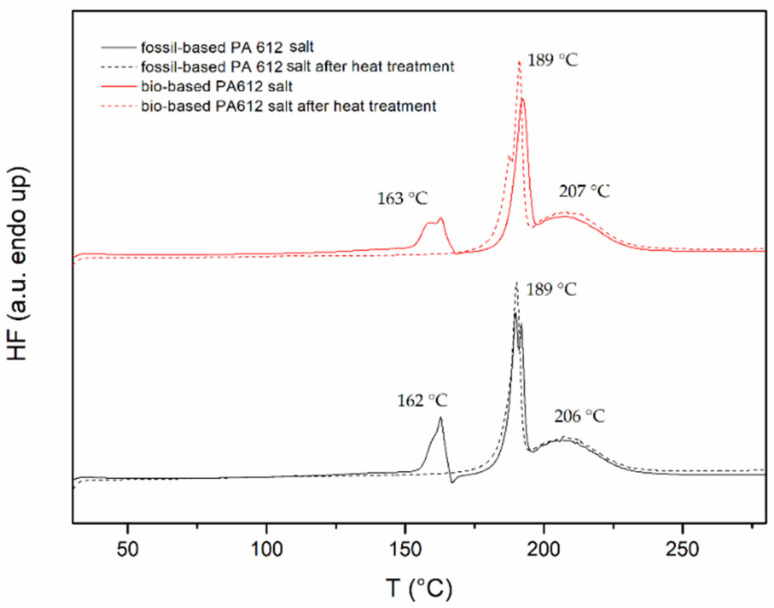
DSC graphs of fossil- and bio-based PA 612 salts.

The thermal properties of the salts prepared were studied. Based on DSC analysis, it was observed that both salts have similar melting temperatures at 189 °C (Table 3). This value is very close to that found in the pertinent work of Boussia et al. (*T_m_* = 185 °C) [25]. On the other hand, the DSC curves revealed that following PA 612 salts melting another broad endothermic peak appears at higher temperatures (Figure 2) This broad peak corresponds to the subsequent melt polymerization of the salt in harmony with PA 66 salt, as an example [28].

In the DSC curves of both grades, it was also possible to observe an additional endothermic peak at 162–163 °C for both PA 612 salt grades, which, however, disappears after heat treatment (Figure 2). This behavior has been also observed before in the case of other aliphatic polyamide salts attributed to the accommodation of a polar solvent (i.e., ethanol, water) in the crystal lattice arrangement during salt formation [44].

**Figure 3:**
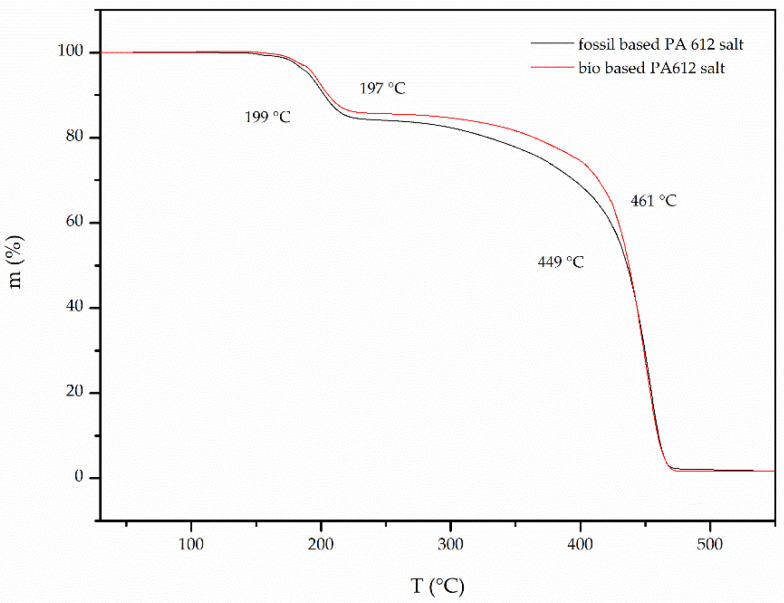
TGA graphs of fossil- and bio-based PA 612 salts.

Focusing more to the TGA curves of both grades (Figure 3), one can observe two mass loss steps: the first step (*T_step1_*) occurred approximately at 170–230 °C with a maximum loss at 197 °C. This step can be attributed to melting and the subsequent polymerization of the salt. The evaporation of the polycondensation water during the reaction leads to the residue recorded on the TGA graphs (84% for the fossil-based and 83% for the bio-based PA 612 salt). To these losses an additional loss of the volatile diamine preceding the polymerization must be added. In other words, the mass loss recorded in the first step, exceeds the theoretical mass loss (equal to 10.4%), attributed to removal of polycondensation water and calculated stoichiometrically by Equation (3):

(3) $$m_{H_{2}O=\frac{2\;\times \;{\mathrm{Mr}}_{H_{2}O}}{{\mathrm{Mr}}_{\mathrm{salt}}}}$$

Finally, the second loss step (*T_step2_*) occurs at approximately 450–460 °C and is attributed to the thermal degradation of the polymer formed during the TGA run [25,28].

**Figure 4:**
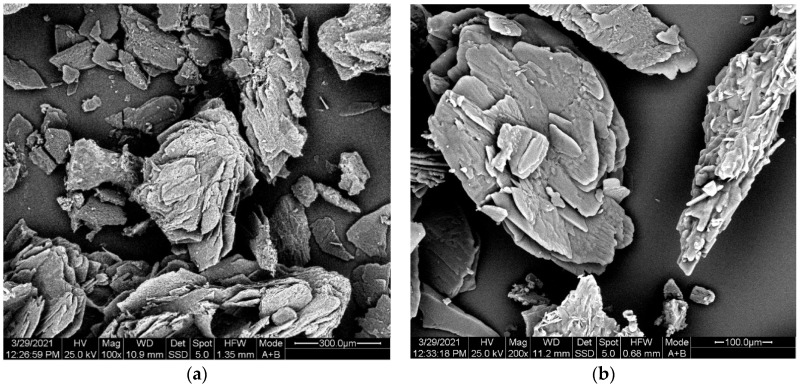
SEM images of PA 612 salt. (**a**) magnification 100× and (**b**) magnification 200×.

Finally, the morphology of the fossil salt crystals was examined via SEM microscopy (Figure 4). For both magnifications used, crystals were characterized by sharp edges and smooth surfaces.

### 3.2. DSSP in Micro Scale

#### 3.2.1. TGA Runs

The DSSP temperature in micro scale runs was fixed at 160 °C, 165 °C, 168 °C and 170 °C, i.e., 20–30 °C below the salt melting point), also to avoid as much as possible the appearance of solid–melt transition (Section 2.3.1 and Table 1).

It must be noted here that at the end of the DSSP runs, the morphology of the final product was always carefully examined, at least macroscopically. For the fossil-based PA 612 salt, samples polymerized at 160 °C and 165 °C maintained the solid state and were received in the form of solid crystals. Samples polymerized at 168 °C and 170 °C fell completely in the molten state and were received as a bulk material. 

Turning to the partly bio-based PA 612 salt, samples polymerized at 160 °C and 165 °C retained macroscopically the solid state while in samples polymerized at 168 °C partial melting and agglomeration was noticed. Finally, samples polymerized at 170 °C melted completely and were received as a bulk material similarly to the fossil-based analogue. 

**Figure 5:**
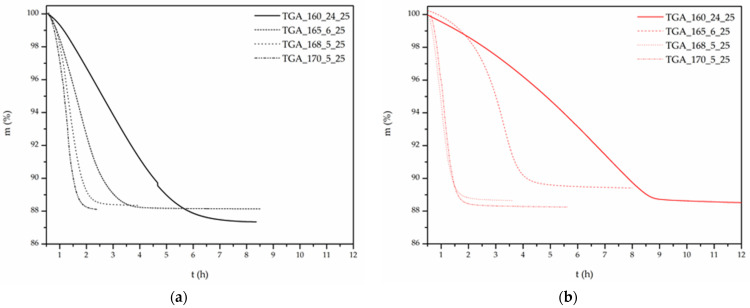
DSSP curves of (**a**) fossil-based and (**b**) bio-based PA 612 salt.

The nature of the salt, fossil- vs. bio-based, has been found to also play a significant role in reaction kinetics (Figure 5, Table 4). Under the same experimental conditions, the fossil-based PA 612 salt has been found to react at a higher rate than the bio-based salt. For example, at 165 °C, the fossil-based salt reaches plateau at 4 h and exhibits a t_1/2_ value at 1.8 h, while at the same temperature the bio-based salt reaches plateau at 5 h and exhibits a t_1/2_ value at 3.1 h. 

The aforementioned difference is in complete harmony with the noticed lag in the appearance of the SMT as discussed above, verifying once more the mechanism suggested for correlating the SMT phenomenon with the accumulation of the water formed during the reaction, i.e., the faster the reaction the earlier the SMT [4,5].

**Figure 6:**
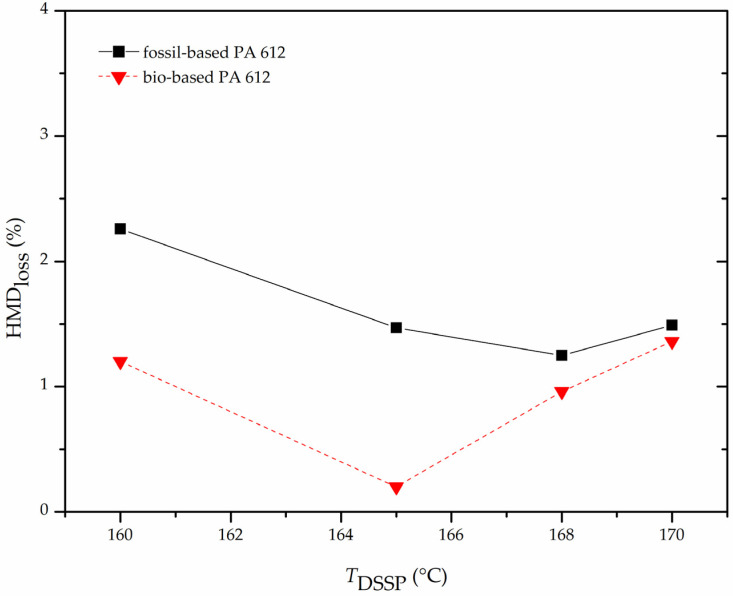
DSSP diamine loss for fossil-based and bio-based PA 612 salt.

Turning to the additional loss of the volatile diamine during the polymerization, diamine loss curves versus reaction temperature (Figure 6) were found to follow a “U” shape for both monomer grades. Higher loss values at low temperatures are observed, then reaching a minimum, and increasing again with increasing reaction temperature. Obviously, in the regime of the low temperatures longer reaction times are required together with concurring amine loss. At the other end, at high temperatures, amine sublimation increases too. At the intermediate region amine loss seems to be “optimized”.

It must be emphasized here that the reaction temperature is a very critical parameter in DSSP as it influences significantly both reaction mechanism and rate [2,3,12,32,33]. As has been observed for the fossil-based PA 612 salt, a small temperature increase from 165 °C to 168 °C leads the reaction to fully fall to the melt state, changing completely the reaction mechanism. Interesting to note is that a 5 °C rise from 160 °C to 165 °C leads to a 42% decrease in t_1/2_. This decrease in t_1/2_ is even higher (61 %) when increasing the temperature to 170 °C but here the reaction kinetics/mechanism is different as the reaction falls to the melt state. A similar effect on reaction mechanism, has been observed also for a 3 °C temperature increase (from 165 °C to 168 °C) for the bio-based PA 612 salt (DSSP to partial melting). Finally, Papaspyrides et. Al, studying the DSSP of the PA 126, noticed the same performance, together with solid–melt transition, for only 2 °C temperature increase (126 °C to 128 °C) [2,3].

#### 3.2.2. Characterization of TGA Products

In all runs the formation of a polyamide structure was confirmed through FT-IR analysis (Figure 1). More specifically, all spectra included the absorptions at approximately 3300 cm^−1^ (hydrogen-bonded–NH stretching vibration–Amide Band I), at approximately 3062 cm^−1^ (Amide B overtone of Amide II), at 1640 cm^−1^ (–C–CO stretching vibration–Amide Band I), at 1540 cm^−1^ (–CN stretching vibration and CONH bend–Amide Band II), at 940 cm^−1^ (–C=O stretching vibration–Amide Band IV), at 690 cm^−1^ (–NH out of plane bend) and at 586 cm^−1^ (–CO out of plane bend) [25,45,46,47,48].

Turning to thermal properties, the melting points of the formed polyamides are in the range from 212 to 214 °C for the fossil-based samples and from 214 to 217 °C for the bio-based ones. These values are slightly lower than commercial samples being in the range of 217–220 °C. It is worth noting that bio-based samples exhibit slightly higher melting points than fossil based while crystallinity values show no significant difference (23–24% for the fossil-based samples and 20–21% for the bio-based samples).

**Figure 7:**
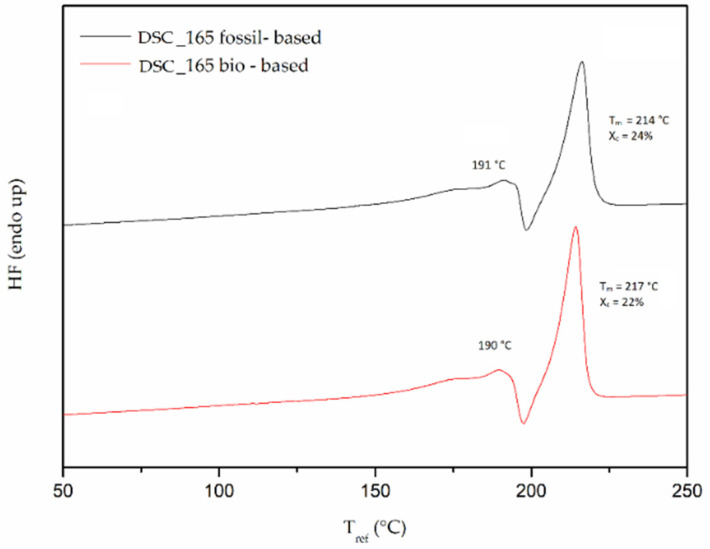
DSC curves of microscale DSSP products at 165 °C.

Typical DSC curves for both grades are given in Figure 7. All samples present a broad endotherm at (190–200 °C) which corresponds to the melting of defective or metastable crystals. This is followed by a small exothermic crystal rearrangement. Finally, at 212–217 °C a sharp endotherm corresponds to the melting of the polyamide. This behavior has been published already and is common for long chain aliphatic polyamides [25,46,47,48,49].

Finally, degradation temperatures are increased for all biobased samples (445–449 °C) when compared to fossil-based ones (439–445 °C), and the same trend is followed for *T_d,5%_* (378–395 °C for bio-based compared to 372–378 °C for fossil-based). These values, while demonstrating good thermal stability, are significantly lower than values presented in literature which are around 428 °C for *T_d,5%_* and 460 °C for degradation temperature [25]. Shorter molecular size might be correlated to this observation.

### 3.3. DSSP in Laboratory Scale

The aforementioned microscale polymerizations (discussed in Section 3.2.1) were carried out in laboratory scale in an attempt to avoid SMT during the reaction but also to achieve a reasonable reaction rate. Based on the pertinent findings the reaction temperature was set at 160, 165 and 168 °C; however, taking into account that only the bio-based salt survived complete melting at 168 °C.

#### 3.3.1. Effect of Reactor Design: Scouting Experiments

The goal here was to compare the two different reactors in terms of maintaining the solid character of the reacting mass during the course of the reaction and bypassing SMT phenomena.

Both fossil and bio-based salts were tested. All experiments were run at the same experimental conditions, namely at 165 °C and for 6 h (experiments R1_165_6_20 and R2_165_6_20, Table 1). Difference in scale was 1200 and 600 times larger for reactors R1 and R2 respectively.

Nitrogen flow was set to 20 mL min^−1^ close to that in the TGA chamber (25 mL min^−1^). However, very different flowing conditions apply as in the TGA runs the nitrogen flows above the aluminum pan with the hole on the cover [28,29] whereas in R1 and R2 nitrogen passes through the reacting mass.

Turning to the results it was clear, even macroscopically, that Reactor 1 products, for both grades, had been passed through a melt state. This contradicts microscale results, as products polymerized there, at 165 °C, maintained the solid state. On the contrary, in Reactor 2 products, for both salt grades and at 165 °C the morphology of the crystals was maintained. This will be discussed in detail in Section 3.3.2. Thus, it has been proved again here the critical role of the reactor design in DSSP [12,24] since all other reaction conditions/critical parameters were the same. In other words, in Reactor 1 DSSP proceeds clearly through melt intermediate stages, even at reaction temperatures much lower than the salt melting point, as has been demonstrated by Papaspyrides et al. [2,3,4,5,6,7,12,17,24]. On the contrary, in Reactor 2, no distinct transition to the melt state occurs, leading us to believe that DSSP proceeds in true solid state [7,17,24,28,29,30].

FT-IR analysis (Figure 1) confirmed polymer formation as the Amide Band I and Amide Band II absorptions are apparent in the spectra for all products from both reactors. 

**Figure 8:**
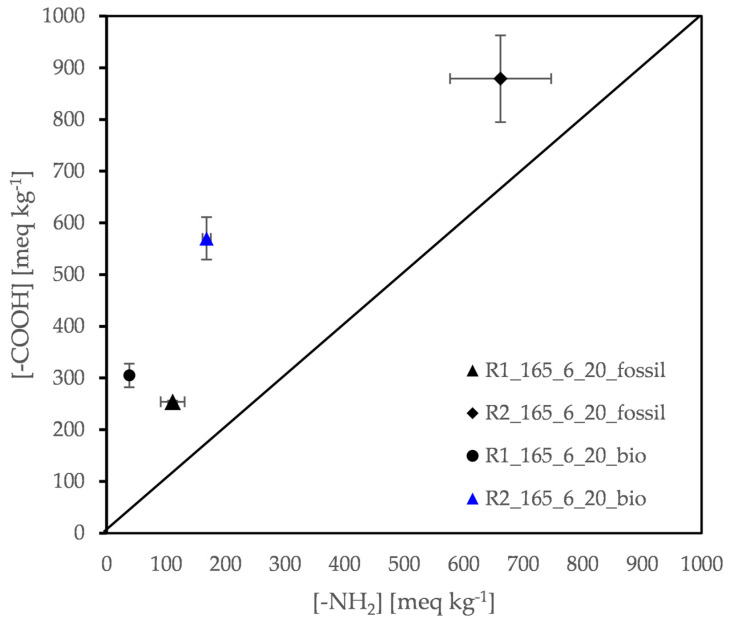
Effect of reactor design: amine and carboxyl end-group concentration of R1 and R2 products.

Turning to the molecular size of the DSSP products, in Table 5 the calculated number average molecular weight $\bar{M_{n}}$ is quoted, based on both end-group concentrations presented graphically in Figure 8. Intrinsic Viscosity ([*η*]) data are also given for comparison.

A considerable difference in performance between the two alternative reactors is noticed. For Reactor 1 the molecular weight achieved for both grades is higher as shown also from the viscosity data (Table 5, Figure 8). In fact, the reaction has proceeded with a higher rate in the case of the R1 Reactor due to the appearance of an intermediate melt stage accelerating conversion, i.e., where a fast melt kinetics mode prevails. This is also due to the better imbalance between amine and carboxyl groups *D* in comparison with Reactor 2. 

In the Experimental Part it has been mentioned that: “Reactor 2 is a fixed bed reactor more capable of removing the water formed”. However, this design also facilitates diamine escape. In fact, HMD vapor pressure (200 Pa at 50 °C), is particularly high at 40 °C below its boiling point (205 °C) and therefore the amine tendency to switch to the gas phase dominates the process. In other words, *D* becomes larger in R2 leading to lower conversion (Table 6). Thus, maintenance of the solid state according to the predominating nucleation and growth model is observed at the expense of conversion [8,9].

As to their thermal properties (Table 6), Reactor 1 products compared with those of Reactor 2 show similar values while DSC curves present double melting phenomena as has been observed in the microscale experiments (and to be discussed again in Section 3.3.2.1).

#### 3.3.2. DSSP in Reactor 2

##### 3.3.2.1. Evaluation of Time–Temperature Profiles Tested

The performance of Reactor 2 was further studied in the range of 160–168 °C following time–temperature profiles extracted from the microscale data (Table 1). Thus, time was varied in the range of 5 to 24 h with decreasing trend as temperature was increased. Nitrogen flow was set in all runs at 20 mL min^−1^. Both fossil-and bio-based salts were tested for comparison. The goal was to optimize DSSP reaction in Reactor 2, always in terms of molecular size while maintaining the solid state. In this context, for both fossil- and bio-based salts the following behavior was observed: 

At 160 °C for 24 h the reaction proceeded clearly in the solid state. The same applies at 165 °C for 6 h and at 168 °C for 5 h. The latter behavior at 168 °C is in agreement with the TGA runs but only for the bio-based salt while the fossil one melted completely (Section 3.2.1). This can be correlated again to the fact that SMT is avoided at the expense of conversion as the TGA t_1/2_ data clearly confirm (Table 4). In conclusion the Reactor 2 seems to play a very critical role in maintaining the solid state.

**Figure 9:**
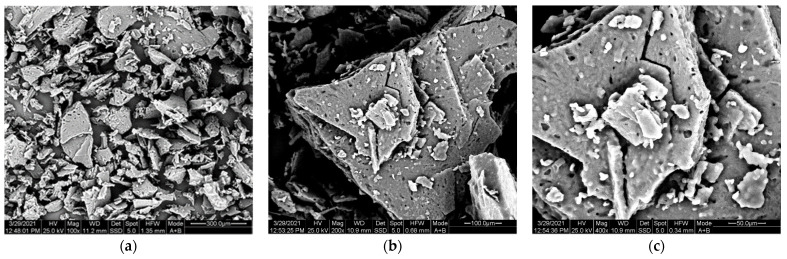

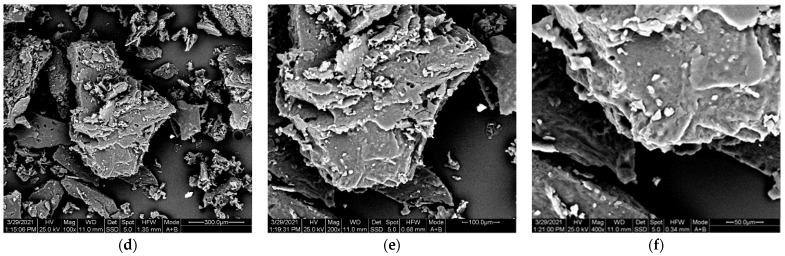
SEM images of R2_165_6_20 samples (**a**–**c**) and PA612_168_5_20 samples (**d**–**f**).

The aforementioned morphology observations at 165 °C and 168 °C were further supported via SEM microscopy. The more “sensitive” fossil-based products were considered here (Figure 9) in comparison with Figure 4 where the starting materials were examined. In all cases and at all magnifications employed the maintenance of the solid state is evident. Equally important all particles present sharp edges while a variety of cracks and holes is observed on the surface of the crystals, in contrast with the smooth surface of the starting material (Figure 4). Obviously, the latter is due to the gradual release of the polycondensation water before SMT.

**Figure 10:**
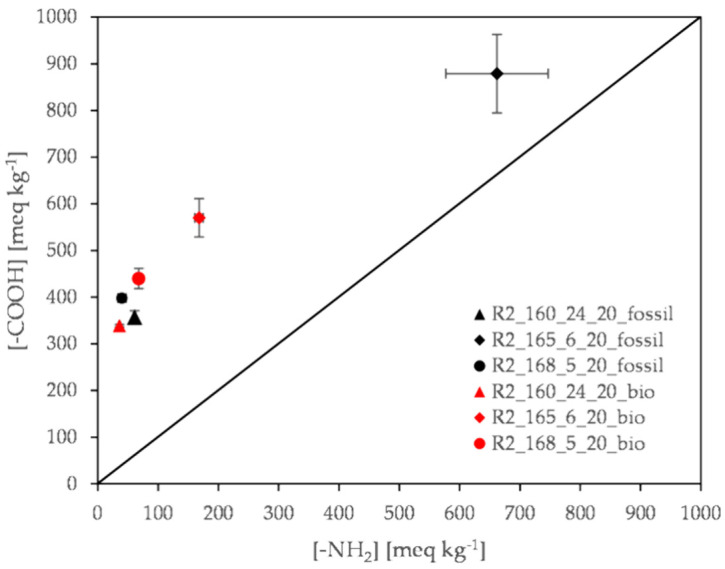
Evaluation of time–temperature profiles tested: amine and carboxyl end group concentration of R2 products.

Turning to the end-group analysis the primary data are given graphically in Figure 10. The molecular size is expressed as number average molecular weight ($\bar{M_{n}}$), in the range of 1300 to 5300 g mol^−1^ (Table 7). More importantly, both Table 7 and Figure 10 show that the end-groups imbalance *D* is minimized at 165 °C for the fossil-based products thus ensuring further reaction if needed. On the other hand, the highest molecular weight for both grades is obtained at 160 °C (4800 g mol^−1^ for the fossil-based PA 612 and 5300 g mol^−1^ for the bio-based) due to the much higher reaction time (24 h compared to 5 or 6 h at 165 or 168 °C respectively). For the shorter time runs the molecular weight increases significantly by increasing the reaction temperature only by 3 °C, as it is demonstrated by the calculated $\bar{M_{n}}$ (from 1300 to 4600 g mol^−1^ for the fossil-based PA612 and from 2700 to 3900 g mol^−1^ for the bio-based one), due to higher reaction rate. In other words, it is confirmed again that the DSSP of nylon salts have a “very high temperature coefficient” as already discussed in Section 3.2.1. Finally, viscosity data confirm the discussion.

DSC spectra of the obtained products present double melting phenomena as has been already discussed in the micro scale runs (Section 3.2.2). As to their melting point (Table 8), no significant difference between the alternative PA 612 grades was observed as all samples exhibit melting points around 217–220 °C, which are comparable to literature and commercial values. On the other hand, crystallinity values remain in the range 18 to 27% while for commercial fossil-based polyamides typically do not exceed 40% and for bio-based polyamides do not exceed 30% [50,51]. This might be attributed to the lower molecular weight of our polyamides.

##### 3.3.2.2. Effect of Nitrogen Flow Rate

It is a well-known fact that in DSSP and post-SSP of polyamide salts the nitrogen flow rate is a critical parameter for ensuring an optimum end group balance. In this study, both reactors R1 and R2 were first purged with nitrogen and then a continuous flow rate at 20 mL min^−1^ was set throughout the whole reaction. However, it has been referred already in literature that, for the DSSP of PA 6T and PA 4T salts, some runs have been carried out at zero flow rate in an effort to reduce diamine loss and avoid SMT [28,30]. The same approach has been tested here modifying the polymerization run [R2_165_6_20] (Table 5 or Table 7) with a new one: [R2_165_6_0], i.e., stopping nitrogen flow rate after purging. It is worthwhile to note that the products of the chosen run showed a minimization of *D*, thus permitting continuation of the reaction in a second stage if needed.

The results, first on maintaining the solid state, revealed that both fossil- and bio-based products provided a similar macroscopic picture at 20 or 0 mL min^−1^ flow rate. Some partial melting close to the reactor walls was only observed [16].

**Figure 11:**
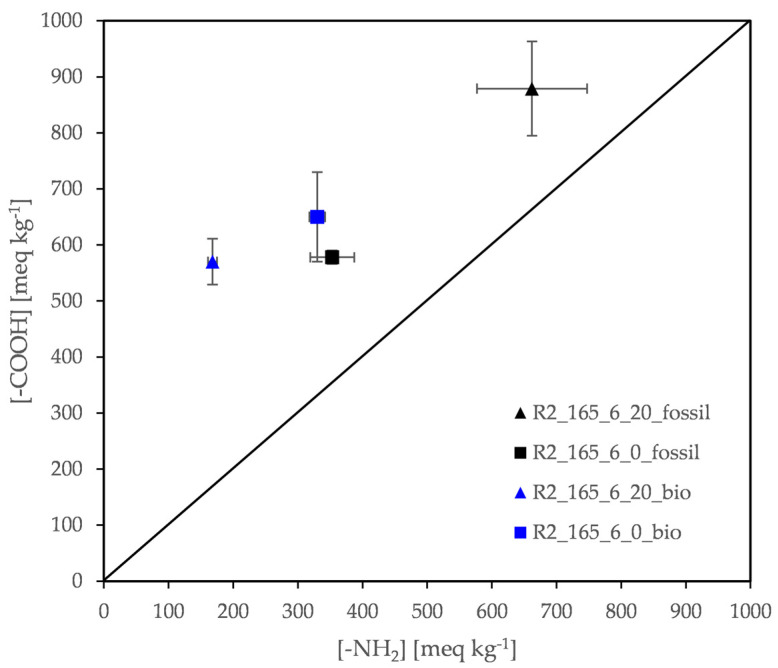
Effect of nitrogen flow rate: amine and carboxyl end group concentration of DSSP products.

Turning to the molecular size of the DSSP products, in Table 9 the calculated $\bar{M_{n}}$ are quoted. These data are based on both end-groups concentrations presented graphically in Figure 11. Intrinsic viscosity [*η*] data are also given for comparison. It is very impressive that abandoning the nitrogen flow leads to a very significant increase of the $\bar{M_{n}}$ and [*η*], while *D* decreases however slightly. This behavior is valid for the fossil-based salt while the qualitative picture provided for the bio-based grade is similar, though not so impressive or consistent.

In the polyamide industry starting from salts, to attain high molecular weights a two-step process is required. A typical example is the commercial production of Nylon 66 [12,36,52,53,54,55,56,57,58,59]: the polymerization proceeds initially in an aqueous solution, then water is removed while the reaction turns to the melt state, followed at the end by the so called “solid state finishing”, i.e., a post SSP step. The latter serves in avoiding unnecessary and harmful exposure of the material at high temperatures. On the other hand, polycondensation processes, mainly on polyesters, are under investigation, based on the same approach: the reaction occurs in steps at increasing temperatures [60].

To apply the aforementioned route in polyamides a critical prerequisite is necessary: the stoichiometric balance between amine and carboxyl groups should remain as close as possible to equimolar conditions, following Flory’s kinetics [61].

In conclusion, the fossil-based PA product under the code conditions [R2_165_6_0] presented the best overall performance, for being the starting material in a subsequent DSSP while testing further Reactor R2.

#### 3.3.3. Demonstration of a Feasible Two-Step DSSP Process

To fulfill the aforementioned prerequisite on *D*, the addition of some amine preceding the post SSP step comprises a widely applied route. This easily corrects D while being very successful in the polyamide industry for decades [30,62,63,64]. In other words, amine addition into already polymerized samples, subjected then to post-SSP, has been proved very attractive. In this context and to demonstrate a feasible industrial process (Table 1), 2% of HMD was added into the [R2_165_6_0] product of fossil-based PA612, being the sample with the lowest end-group deviation as mentioned already. After the amine addition, the sample was polymerized at 170 °C for 4 h again under zero nitrogen flow.

**Figure 12:**
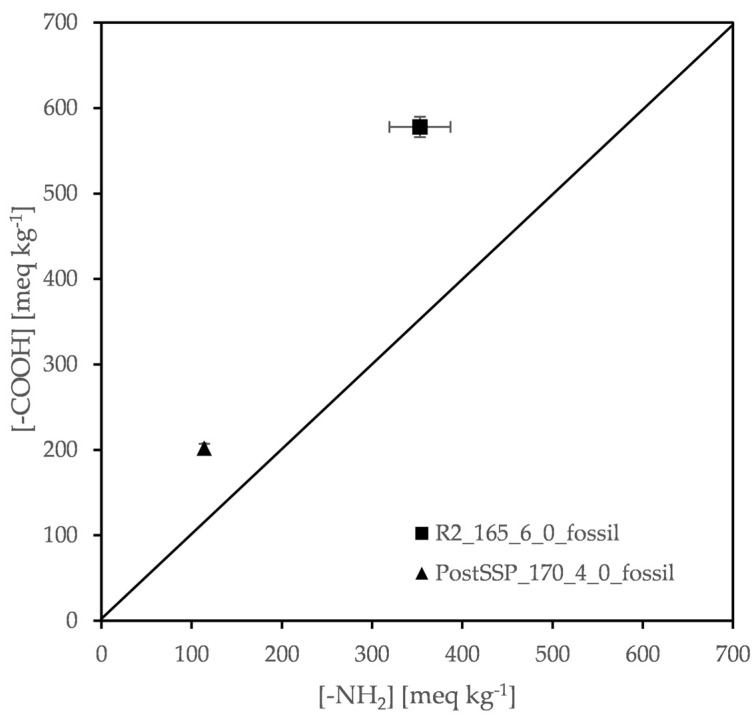
The two-step DSSP process: amine and carboxyl end group concentration of one-step and two-step DSSP products.

The post-SSP product was received as solid free-flowing particles, retaining their original morphology throughout the reaction. Amine addition has been proven successful in increasing the molecular weight of the final product while maintaining a good end-group balance (Figure 12). In particular, a 17% increase in intrinsic viscosity was reported, while the number average molecular weight almost tripled to 6300 g mol^−1^ (Table 10). Finally, the final product contained a fair amount of end-groups (114 meq kg^−1^ amine groups and 202 meq kg^−1^ carboxyl groups), meaning that it is suitable for further polymerization. 

Thus, for the first time an aliphatic nylon salt, the hexamethylene diammonium dodecanoate, has been polymerized via direct solid state polymerization, i.e., starting from free-flowing salt crystals and ending to polymer particles. The process itself is environmentally friendly, comprising of two isothermal steps at low temperatures, for short times and at atmospheric pressure. Finally, the design of the Reactor R2, in contrast with a conventional autoclave R1, catalytically contributed to the success of this project.

## 4. Conclusions

The aim of this work was to apply DSSP on aliphatic nylon salts. The case of the PA 612 salt, fossil-based or bio-based, was chosen. The process should bypass the SMT phenomena occurring in DSSP. In this context, emphasis was given first to reactor design, being the critical issue for understanding and controlling inherent kinetics, amine loss and byproduct diffusion. Different time–temperature profiles were tested first at microscale and then at laboratory scale via two alternative reactors. Occurrence of SMT, when scaling up from microscale to a conventional laboratory autoclave, was in harmony with similar studies on numerous aliphatic nylon salts. The picture changed when using the new laboratory reactor. A series of scouting experiments on the critical reaction parameters concluded to the demonstration of a real DSSP process, starting from crystals of PA 612 salt and ending to PA 612 particles without any intermediate melt stages. Two isothermal steps, while correcting amine end-groups content between, have been involved. Finally, the fossil-based PA 612 salt provided better end-groups imbalance than the bio-based salt.

## Figures and Tables

**Table 1:** DSSP reaction conditions and nomenclature for all experiments.

	*T*_DSSP_ (°C)	*t* (h)	N_2_ Flow (mL min^−1^)
**Micro Scale DSSP**			
**TGA_160_24_25**	160	24	25
**TGA_165_6_25**	165	6	25
**TGA_168_5_25**	168	5	25
**TGA_170_5_25**	170	5	25
**Laboratory Scale DSSP**			
*Effect of Reactor Design: Scouting Experiments*			
**R1_165_6_20**	165	6	20
**R2_165_6_20**	165	6	20
*Evaluation of Time-Temperature Profiles Tested*			
**R2_160_24_20**	160	24	20
**R2_165_6_20**	165	6	20
**R2_168_5_20**	168	5	20
*Effect of Nitrogen Flow Rate*			
**R2_165_6_20**	165	6	20
**R2_165_6_0**	165	6	0
**Demonstration of a Feasible Two-Step DSSP**			
**R2_165_6_0**	165	6	0
**Post_SSP_170_4_0**	170	4	0

**Table 2:** End-group concentrations of PA 612 salts.

	[NH_3_^+^]_theor_ = [COO^−^]_the__ο__r_ (meq kg^−1^)	[COO^−^] (meq kg^−1^)	[NH_3_^+^] (meq kg^−1^)	D = [COO^−^] − [NH_3_^+^] (meq kg^−1^)
**Fossil-based PA 612 salt**	5772	5740 ±110	5680 ± 90	60
**Partly biobased PA 612 salt**	5772	5790 ± 100	5670 ± 22	120
**Boussia et al. PA612 salt** [25]	5772	5580 ± 100	5720 ± 60	−140
**Commercial PA 66 salt**	7620	8170 ± 170	7650 ± 170	520

**Table 3:** Thermal properties of PA 612 salts (DSC single heating, TGA).

	*T_m_* (°C)	Δ*H_m_* (J g^−1^)	*T_step1_* (°C)	*T_step2_* (°C)	m_step_1_ (%)
**Fossil-based PA 612 salt**	189	167	199	449	84
**Bio-based PA 612 salt**	189	175	197	461	83

**Table 4:** Mass loss and t_1/2_ for the DSSP of fossil-based and bio-based PA 612 salt.

Salt Grade	*T*_DSSP_ (°C)	m_loss_ (%)	t_1/2_ (h)
**Fossil-based PA 612 salt**	160	12.7	3.1
	165	11.9	1.8
	168	11.7	1.4
	170	11.9	1.2
**Bio-based PA 612 salt**	160	11.6	5.3
	165	10.6	3.1
	168	11.3	1.0
	170	11.8	1.1

**Table 5:** Effect of reactor design: molecular size and end-group imbalance of R1 and R2 products.

	[*η*] (dL g^−1^)		$\bar{M_{n}}$ (kg mol^−1^) *		D = [COOH] − [NH_2_] (meq kg^−1^)	
	fossil	bio	fossil	bio	fossil	bio
**R1_165_6_20**	0.713 ± 0.010	0.684 ± 0.021	5400	5800	143	267
**R2_165_6_20**	0.251 ± 0.020	0.387 ± 0.021	1300	2700	217	402

* $\bar{M_{n}}$ was calculated by end-group determination.

**Table 6:** Effect of reactor design: thermal properties of R1 and R2 products.

	*T_m_* (°C)		Δ*H_m_* (J g^−1^)		*X_c_* (%)		*T_d_* (°C)		*T_d,5%_* (°C)		Residue (%)	
	fossil	bio	fossil	bio	fossil	bio	fossil	bio	fossil	bio	fossil	bio
**R1_165_6_20**	216	213	53	69	20	27	460	475	378	378	3	3
**R2_165_6_20**	217	217	48	58	18	22	457	454	378	379	3	3

**Table 7:** Evaluation of time–temperature profiles tested: molecular size and end-group imbalance of R2 products.

	[*η*] (dL g^−1^)		$\bar{M_{n}}$ (kg mol^−1^) *		D = [COOH] − [NH_2_] (meq kg^−1^)	
	fossil	bio	fossil	bio	fossil	bio
**R2_160_24_20**	0.480 ± 0.002	0.610 ± 0.006	4800	5350	296	303
**R2_165_6_20**	0.251 ± 0.020	0.387 ± 0.021	1300	2700	217	402
**R2_168_5_20**	0.410 ± 0.035	0.475 ± 0.016	4550	3950	358	372

* $\bar{M_{n}}$ was calculated by end-group determination.

**Table 8:** Evaluation of time–temperature profiles tested: thermal properties of R2 products.

	*T_m_* (°C)		Δ*H_m_* (J g^−1^)		*X_c_* (%)		*T_d_* (°C)		*T_d,5%_* (°C)		Residue (%)	
	fossil	bio	fossil	bio	fossil	bio	fossil	bio	fossil	bio	fossil	bio
**R2_160_24_20**	220	217	50	69	19	27	455	448	374	378	3	3
**R2_165_6_20**	217	217	48	58	18	22	457	454	378	379	3	3
**R2_168_5_20**	209	218	58	48	22	19	453	448	376	380	2	3

**Table 9:** Effect of nitrogen flow rate: molecular size and end-groups imbalance of DSSP products.

	[*η*] (dL g^−1^)		$\bar{M_{n}}$ (kg mol^−1^) *		*D* = [COOH] − [NH_2_] (meq kg^−1^)	
	fossil	bio	fossil	bio	fossil	bio
**R2_165_6_20**	0.251 ± 0.020	0.387 ± 0.021	1300	2700	217	402
**R2_165_6_0**	0.448 ± 0.020	0.433 ± 0.085	2150	2050	208	320

* $\bar{M_{n}}$ was calculated by end-group determination.

**Table 10:** The two-step DSSP process: molecular size and end-group imbalance of one-step and two-step DSSP products.

	[*η*] (dL g^−1^)	$\bar{M_{n}}$ (kg mol^−1^) *	*D* = [COOH] − [NH_2_] (meq kg^−1^)
Fossil—based			
**R2_165_6_0**	0.448 ± 0.020	2100	208
**Post_SSP_R2_170_4_0**	0.522 ± 0.015	6350	87

* $\bar{M_{n}}$ was calculated by end-group determination.

## Data Availability

Data is contained within the article.
